# Model-informed AUC-guided dosing for parenteral vancomycin in critically ill patients: impact on target attainment and renal safety – a pre-post intervention study

**DOI:** 10.1186/s40360-026-01224-x

**Published:** 2026-09-15

**Authors:** Laura Gundlach, Mareike Kunkel, Oliver Scherf-Clavel, Güzin Surat

**Affiliations:** 1 Pharmacy, University Hospital Wuerzburg, 97076 Wuerzburg, Germany; 2 Unit for Infection Control and Antimicrobial Stewardship, University Hospital Wuerzburg, 97080 Wuerzburg, Germany; 3 Department of Pharmacy, Ludwig-Maximilians-University, Clinical Pharmacy and Pharmacotherapy, 81377 Munich, Germany

**Keywords:** AKI, Antimicrobial stewardship, AUC_24_-based dosing, MIPD, MRSA, Non-MRSA, Target attainment, Vancomycin

## Abstract

**Background:**

Parenteral vancomycin is essential for the treatment of certain gram-positive infections but is associated with acute kidney injury (AKI). The 2020 IDSA guideline recommends AUC_24_-based dosing for MRSA to increase treatment safety. Due to the low MRSA prevalence at the University Hospital of Würzburg (UKW), this study investigated the impact of model-informed precision dosing (MIPD) of vancomycin on AKI incidence and target attainment in MRSA and non-MRSA infections.

**Methods:**

This pre-post intervention study compared a retrospective group (January 1, 2019 - June 30, 2021) with a prospective group (October 17, 2022 - December 31, 2023), including critically ill patients from intensive and intermediate care units. The prospective group received AUC_24_-based MIPD using Bayesian pharmacometric modeling performed by a clinical pharmacist. The primary outcome was the incidence of AKI. Secondary outcomes included vancomycin therapeutic target attainment and the proportions of therapies within, below, and above the therapeutic range.

**Results:**

AKI occurred in 27.8% of the retrospective group and 14.0% of the prospective group (*p* = 0.04). The AUC-based group achieved therapeutic targets more frequently (70.2% vs. 55.1%, *p* = 0.049) and had fewer supratherapeutic levels (8.8% vs. 24.5%, *p* = 0.01).

**Conclusions:**

AUC_24_-based MIPD for vancomycin improved therapeutic target attainment and was associated with a lower observed incidence of AKI. These findings support further evaluation of AUC-guided dosing for both MRSA and non-MRSA infections.

**Supplementary Information:**

The online version contains supplementary material available at https://doi.org/10.1186/s40360-026-01224-x.

## Background

The glycopeptide antibiotic vancomycin is a broad-spectrum antibiotic against gram-positive pathogens and is administered parenterally for the empiric and definitive management of infections caused by streptococcal, staphylococcal (e.g., methicillin-resistant *Staphylococcus aureus or Staphylococcus epidermidis*), or enterococcal (e.g., *Enterococcus faecium*) species resistant to β-lactam antibiotics. The use of vancomycin is associated with various adverse events like infusion-related reactions, ototoxicity and an increased risk of acute kidney injury (AKI), leading to prolonged hospitalization, higher morbidity, and increased healthcare costs [1–4]. To minimize the risk of AKI, the corresponding IDSA guideline “Vancomycin Therapeutic Guidelines: A Summary of Consensus Recommendations from the Infectious Diseases Society of America, the American Society of Health-System Pharmacists, and the Society of Infectious Diseases Pharmacists” from 2009, recommends close monitoring of vancomycin trough levels, measured at steady state, with target concentrations based on the severity and complexity of the infection. In order to avoid treatment failure and reduce the risk of adverse effects a minimum of a trough level of > 10 mg/L with a therapeutic range of 15–20 mg/L is advised [5]. In March 2020, the American Society of Health-System Pharmacists, the Infectious Diseases Society of America, the Pediatric Infectious Diseases Society and the Society of Infectious Diseases Pharmacists published a guideline on optimizing the monitoring and dosing of vancomycin in methicillin-resistant *Staphylococcus aureus* (MRSA) infections [6]. Rather than relying on trough concentrations, the guideline suggests targeting an area under the curve (AUC), which delivers a more informative measure of drug exposure, since it does not rely on a single point of the pharmacokinetic curve like trough levels. The guideline advocates an AUC target of 400–600 mg⋅h/L over 24 h, provided that the minimum inhibitory concentration (MIC) does not exceed 1 mg/L. The conservative method of measuring trough levels involves only a single serum concentration, but calculating the AUC_24_ is a more complex pharmacometric task. The guideline outlines several methods for calculating the AUC_24_/MIC. The option favored by the guideline is the use of Bayesian methods to determine the AUC over 24 h. This approach combines complex population pharmacokinetic knowledge, so-called a priori knowledge, with the patient’s dosing history and patient characteristics to create new a posteriori knowledge, also known as model-informed precision dosing (MIPD). One of the major challenges in implementing MIPD is the selection of a suitable pharmacokinetic model. If the chosen model is not representative of the individual patient or the clinical setting, significant differences in predicted drug levels can result. Numerous models exist for vancomycin dosing for different patient populations, making careful model selection essential to ensure an appropriate fit. Methods such as model averaging or automated model selection can improve this issue by pooling information across models or identifying the most suitable model for the specific patient [7–11]. The prevalence of MRSA colonization and infections at the University Hospital of Würzburg is low (refer supplementary table S1), as it is in Germany in general (refer supplementary table S2) [12]. However, parenteral vancomycin is considered empirically for infections associated with foreign materials, such as prosthesis infections, or postoperative wound complications, including mediastinitis, spondylodiscitis or postoperative meningitis caused by methicillin-resistant staphylococci. Therefore, the wider use of dose optimization according to the guideline is warranted by the need to secure the safest possible therapy for all patients. Furthermore, subtherapeutic levels are not only associated with treatment failure but also promote the development of antimicrobial resistance, highlighting the importance of dose optimization for all vancomycin therapies regardless of the pathogen [4, 5, 13]. To the best of our knowledge, the guideline has primarily been executed in Germany by adopting the approach of recording two concentrations and utilizing first-order pharmacokinetic equations to estimate the AUC. At the University Hospital of Würzburg (UKW), the antimicrobial stewardship team introduced this relatively new pharmacometric AUC_24_-based dosing of vancomycin and decided to make use of this model irrespective of the indication for vancomycin. Therefore, this study evaluated whether implementation of Bayesian AUC-guided model-informed precision dosing improves renal safety and therapeutic target attainment in a real-world cohort predominantly comprising patients with non-MRSA infections. Specifically, the incidence of AKI before and after implementation was assessed together with therapeutic target attainment und the distribution of subtherapeutic, therapeutic, and supratherapeutic drug exposure. Furthermore, the impact of potential covariates on the safety of vancomycin treatment such as concomitant diseases (obesity, diabetes mellitus) or potentially nephrotoxic concomitant medication (aminoglycosides, loop diuretics, piperacillin/tazobactam) was systematically analyzed.

### Patients and methods

The study was designed as a pre-post intervention investigation comparing a retrospective with a prospective group. The retrospective group was a single-center patient collective whose data were obtained from the electronic patient record of the respective wards and subsequently analyzed. Patient data were included for a period from January 1, 2019, to June 30, 2021. The prospective data were collected from the same electronic patient record system as the retrospective data, during a period from October 17, 2022, to December 31, 2023, after a gradual switch to pharmacometric AUC_24_-based dosing of parenteral vancomycin had taken place at the University Hospital of Wuerzburg starting in October 2022. In accordance with institutional guidelines, an ethics waiver was granted by the ethics committee of Julius-Maximilians-University Würzburg (20240122 02).

Vancomycin therapies were excluded if covariates (age, sex, weight, height) or comedication were incomplete. Dialysis patients who had undergone dialysis before or at the start of vancomycin therapy were excluded from further analysis. The retrospective patient population included all individuals with at least one documented vancomycin level and a minimum of two documented vancomycin administrations. Patients who required dialysis for the first time at least 24 h after initiation of vancomycin therapy remained eligible for inclusion to avoid excluding patients who developed AKI requiring dialysis during the observation period. The retrospective data analysis included only patients from intensive and intermediate care wards due to the ongoing roll-out of the electronic patient record for general wards, which prevented electronic data retrieval in these wards. The prospective group included all patients who received parenteral vancomycin while being treated in one of the participating intensive care or intermediate care units associated with the project. Seven intermediate and intensive care wards (e.g., intensive care unit for anesthesia, cardiothoracic surgery, neurosurgery, general surgery, and internal medicine) were included in the project. Inclusion criteria for the prospective group required at least one documented vancomycin level, a minimum of two vancomycin doses and at least one dosing recommendation provided by a pharmacist. Missing patient covariates were obtained through telephone inquiries to the respective wards, allowing these patients to be included in the analysis. For comparability with the retrospective group, only patients receiving treatment in intensive or intermediate care units were encompassed in the analysis. The study included collecting data on patients’ age, sex, weight, baseline serum creatinine levels, and concomitant medications, including piperacillin/tazobactam, loop diuretics (furosemide, torasemide), aminoglycosides (gentamicin, amikacin), amphotericin B, flucloxacillin, non-steroidal anti-inflammatory drugs (ibuprofen, acetyl salicylic acid), vasopressors, antidiabetic drugs (metformin), and immunosuppressants (ciclosporin, tacrolimus, and everolimus). Data extraction and documentation for both groups were performed by the same investigator to minimize potential discrepancies. Comorbidities including obesity, diabetes, and other relevant conditions were also recorded. The primary focus of this investigation was to evaluate the occurrence of AKI before and after the implementation of AUC-based MIPD for vancomycin. AKI was defined according to the Kidney Disease: Improving Global Outcomes (KDIGO) criteria as an increase in serum creatinine of ≥ 0.3 mg/dL within 48 h or an increase to ≥ 1.5-times the baseline value within 7 days [14]. An interrupted time series analysis (ITSA) was also performed to assess the immediate and gradual effects of the intervention on the proportion of therapies associated with AKI. Therapeutic target attainment was evaluated as a secondary outcome. Therapeutic targets were evaluated according to the therapeutic drug monitoring strategy used during the respective study period. In the retrospective cohort, target attainment was assessed using measured vancomycin trough concentrations, reflecting the institutional standard operating procedure for trough-guided dosing. Trough concentrations < 10 mg/L were classified as subtherapeutic, concentrations of 10–20 mg/L as therapeutic, and concentrations > 20 mg/L as supratherapeutic. Following the implementation of AUC-guided dosing, target attainment in the prospective cohort was assessed using Bayesian-estimated AUC_24_-values. AUC_24_-values < 400 mg⋅h/L were classified as subtherapeutic, values of 400–600 mg⋅h/L as therapeutic, and values > 600 mg⋅h/L as supratherapeutic. Target attainment was assessed using the vancomycin trough concentrations (trough-guided cohort) or AUC_24_-value (AUC-guided cohort) obtained closest to 120 h after initiation of vancomycin therapy. The 120-hour time point was selected to allow sufficient time for therapeutic drug monitoring and subsequent dose adjustments during ongoing therapy. The proportions of therapies within, below, and above the respective therapeutic target range after 120 h of treatment were compared between the two cohorts. In addition, AUC_24_-values were retrospectively estimated for the trough-guided therapies by entering the data into the MIPD software. To implement AUC_24_-based MIPD for vancomycin at UKW, a new SOP was developed. This SOP was implemented on the wards participating on the project, primarily those that frequently administer parenteral vancomycin, such as intensive and intermediate care units. Pediatric wards were not part of the project. The SOP recommended the administration of a loading dose of 25–30 mg/kg of actual body weight depending on infection severity, with a maximum loading dose of 3 g, even for obese patients. Owing the Bayesian dosing method, serum vancomycin concentrations could be measured at any time within the dosing interval, independent of the steady state. Dosing information (dose and administration time) was retrieved from the electronic patient records. Before implementation of AUC-guided dosing, vancomycin serum concentrations were measured by the laboratory and reported to the physicians, who were responsible for dose adjustments. Following implementation, the sampling process, laboratory measurements, and reporting remained unchanged. The main difference was the prospective involvement of a clinical pharmacist in the therapeutic drug monitoring process. MIPD was performed by a clinical pharmacist trained in the use of InsightRX and Bayesian pharmacokinetic modelling. Using InsightRX Nova (version 1.43.6, 2023, InsightRX, San Francisco, CA, USA), a clinical pharmacist generated an individualized dosing recommendation based on a suitable population model, patient dosing information, and covariates. InsightRX uses Bayesian maximum a posteriori (MAP) estimation based on published population pharmacokinetic (popPK) models to individualize vancomycin exposure. Patient-specific covariates entered into the software included age, sex, body weight, height, serum creatinine, dosing history, and measured vancomycin concentrations. Bayesian estimation was preferentially performed using two vancomycin serum concentrations obtained at different time points within the same dosing interval. If only a single concentration was available, Bayesian estimation was performed using that concentration. Throughout this study, the modified popPK models by Thomson et al. and by Goti and Tong et al. served as the standard models, whereas the popPK model by Carreno et al. was considered for obese patients [15–18]. In obese patients, the software compared the fit of the popPK model by Carreno et al. with either the modified popPK model by Thomson et al. or the modified popPK model by Goti and Tong et al., and selected the model with the better goodness-of-fit. PopPK model selection was primarily guided by individual patient characteristics and model fit rather than the clinical setting. In clinical routine, the modified popPK models by Goti and Tong et al., and by Thomson et al. were most frequently selected, whereas the popPK model by Carreno et al. was primarily used in obese patients when it demonstrated the better goodness-of-fit [16–18]. Model fit is a parameter that evaluates goodness-of-fit by comparing measured values to individualized best-fit predictions, thereby reflecting the reliability of predictions. Measured values can be weighted using linear last value prioritization or exponential weighting. Adjustments to the weighting of the available data were based on the patient’s clinical stability. If serum creatinine concentrations or the clinical condition changed, the predefined linear weighting was adjusted to prioritize the most recent measurements. These individually calculated recommendations were communicated to the attending physician, either by telephone or in writing. The frequency of level determination was guided by the patient’s clinical situation and was not systematically changed following implementation of the AUC-guided dosing strategy, with critically ill patients, those undergoing dialysis, or those with hemodynamic changes requiring regular monitoring every 24–72 h. This process was continued until therapy was completed. The SOP further specified that a trough concentration should be obtained whenever the therapeutic drug monitoring was required outside the regular working hours of the clinical pharmacist, for example at weekend, so that vancomycin exposure could still be assessed by the attending physicians when AUC-based dose adjustment was not immediately available.

### Statistical analysis

Continuous baseline characteristics were expressed as mean values and compared using a Welch test (unequal variances t-test). Nominal baseline characteristics were expressed as percentages and compared using the χ2 test. The endpoints were compared using a Fisher exact test. For these comparisons, unadjusted Odds Ratios (OR) with 95% confidence interval (CI) were reported. A p-value < 0.05 was considered statistically significant. Time to AKI occurrence was analyzed using Kaplan-Meier estimates, and unadjusted between group differences were tested using the log-rank test. Model fit for Cox hazard regression was assessed using the Likelihood Ratio Test, Wald Test and score (Log-Rank) Test. A good model fit was indicated by p-values < 0.05. Propensity score matching was performed using a nearest neighbor algorithm without replacement, applying a 1:2 matching ratio and a caliper width of 0.2 to balance baseline covariates between groups. Logistic regression was then applied to the matched data to evaluate the association between dosing strategy and risk of AKI, with results expressed as OR and a 95% confidence interval. Hazard ratios (HR) with 95% CI were reported for Cox proportional hazard regression analyses. The interval time series analysis was performed and different model specifications, including ARIMA models, were tested to identify the best fitting approach. A segmented linear regression model was selected based on goodness of fit and diagnostic tests. Autocorrelation and multicollinearity were assessed using Durbin-Watson test and variance inflation factors. Residual diagnostics ensured the validity of the model. All statistical analyses were performed using RStudio 2024.12.0 + 467 (“Kousa Dogwood Release”) including packages “ggplot2”, “dplyr”, “tidyverse”, “devtools”, “lubridate”, “stringr”, “psych”, “openxlsx”, “survival”, “survminer”, “car”.

## Results

This study included 216 patients in the retrospective trough-based dosing group and 57 patients in the prospective AUC_24_-based dosing group (refer Fig. 2a and b). Baseline characteristics, including body weight, height, and gender distribution, were comparable between the two groups (Table 1). The average number of loading doses and the concomitant use of loop diuretics were also consistent across the groups. Concomitant diseases analyzed occurred with the same frequency in both groups. There was also no difference between the average time until the first vancomycin concentration was determined (27.6 h vs. 27.1 h), as confirmed by a Welch test, which returned a p-value of 0.91, indicating no statistically difference between the groups. Differences were observed in age distribution, serum creatinine baseline levels, and duration of treatment. The retrospective group was more likely to receive concomitant treatment with piperacillin/tazobactam, vasopressors, non-steroidal anti-inflammatory drugs, and aminoglycosides. In the retrospective cohort of patients dosed according to trough levels, AKI was observed in 60 of 216 patients (27.8%). In the prospective pharmacometric AUC_24_/MIC group, AKI occurred in 8 of 57 patients (14.0%). The difference between the groups was statistically significant (p-value = 0.04), with an odds ratio of 2.32 [95% CI 1.08–5.59] (refer supplementary table S3). In the unadjusted analysis, Kaplan-Meier estimates showed a shorter time to AKI occurrence in the trough-guided cohort (log-rank test: χ²= 5.6, *p* = 0.02, refer supplementary figure S1). This unadjusted difference was not confirmed after adjustment for baseline differences in the Cox regression and propensity score-matched analyses. To control potential confounding variables, propensity score matching (1:2 nearest neighbor with a caliper of 0.2) was performed based on the covariates including sex, total body weight, age, baseline creatinine, BMI, and the presence of nephrotoxic co-medications (loop diuretics, piperacillin/tazobactam, aminoglycosides, immunosuppressants, NSAIDs, antidiabetics, aciclovir, amphotericin, flucloxacillin). The matching resulted in well-balanced groups, as indicated by standardized mean differences mostly below 0.1. Subsequent analyses on the matched data did not reveal a statistically significant association between AUC-guided dosing and the risk of AKI (χ² test, *p* = 0.71; logistic regression: OR for AUC-guided versus trough-guided dosing = 0.74, 95% CI 0.26–1.9, *p* = 0.54). An exploratory interaction analysis showed no evidence that the association between dosing strategy and AKI was modified by concomitant piperacillin/tazobactam therapy (interaction term HR 3.20, 95% CI 0.65–15.80; *p* = 0.15). Similarly, no significant interaction was observed for concomitant vasopressor therapy (interaction term HR 2.34, 95% CI 0.24–22.79; *p* = 0.46). The ITSA demonstrated an immediate effect (*p* = 0.019) and a gradual effect over time (*p* = 0.0012). The model explained approximately 43.5% of the variance in AKI occurrence (R^2^ = 0.435) and showed no evidence of autocorrelation (Durbin-Watson *p* = 0.9042) (refer supplementary figure S2). Target attainment at day 5 after the initiation of parenteral vancomycin therapy revealed significant differences between the groups (refer supplementary figure S3). The therapeutic range was achieved in 55.1% of therapies in the trough-based group and in 70.2% in the AUC_24_/MIC group (*p* = 0.049). The proportion of therapies in the supratherapeutic range also differed significantly with 24.5% in the retrospective group and 8.8% in the prospective AUC_24_-based group (*p* = 0.01). The proportion of therapies below the therapeutic range was comparable between the groups and did not show a statistically significant difference (refer Table 2, supplementary table S4 and S5). The calculated AUC values showed that 120 h after initiation of treatment, 21.8% of the therapies were in a supratherapeutic range, 41.7% within the target range and 36.3% remained subtherapeutic (refer supplementary table S6). A Sankey diagram illustrates the distribution and changes in target attainment between the analyzed groups over time (refer Fig. 3). A total of 878 and 289 vancomycin concentrations were measured in the trough-based and AUC24/MIC groups. This corresponded to a mean of 4.1 and 5.1 concentrations per therapy. In the prospective AUC24/MIC group, a total of 145 clinical pharmacist’s recommendations were documented during vancomycin therapy. Of these, 84 (57.9%) concerned dose adjustments, while 61 (42.1%) recommended continuation of the current dosing regimen. Overall, 139 (95.9%) recommendations were implemented, while 6 (4.1%) were not implemented (refer Table 3).

**Table 1:** Baseline characteristics of the patients included in the analysis from trough-based and AUC_24_-based dosing group

	TROUGH (*n* = 216)	AUC_24_/MIC ICU (*n* = 57)	*p*-values
Sex, male, n (%)	157 (72.6)	38 (66.7)	0.47
Total body weight [kg]	83.6; 82 (70–93)	83.2; 83 (66–93)	0.90
Height [cm]	173.0; 174 (168–180)	171.8; 175 (165–180)	0.37
Age [years]	65.1; 66 (57.8–74)	57.3; 61 (48–69)	< 0.01
Loading dose, n (%)	129 (59.7)	36 (63.2)	0.75
Serum Creatinine [mg/dL]	1.24; 1.03 (0.73–1.50)	1.0; 0.88 (0.60–1.26)	0.01
Duration of therapy [days]	5.24; 3.95 (2.20–7.07)	6.41; 5.01 (2.96–8.67)	0.08
First measured vancomycin concentration [hours]	27.6; 22.4 (11.3–35.0)	27.1; 18.0 (8.25–38.67)	0.91
Total daily vancomycin dose [mg/day] including loading dose	1637.9; 1625.0 (1175.6–2000.0)	1654.5; 1562.5 (1225.0-2031.7)	0.81
Number of measured vancomycin concentrations per patient	4.1; 3 (1–5)	5.1; 4 (2–6)	< 0.01
Concomitant medications, n (%)			
Piperacillin/Tazobactam	108 (50.0)	17 (29.8)	0.01
Loop Diuretics (Furosemide, Torasemide)	119 (55.1)	35 (61.4)	0.46
Vasopressors	163 (75.5)	37 (64.9)	0.15
NSAID (Ibuprofene, Acetylsalicylic acid)	31 (14.4)	4 (7.0)	0.21
Aminoglykoside (Gentamicin, Amikacin)	22 (10.2)	1 (1.8)	0.06
Immunosuppressants (Ciclosporin, Tacrolimus, Everolimus)	17 (7.9)	11 (19.3)	0.02
Aciclovir	11 (5.1)	7 (12.3)	0.10
Amphotericin B	10 (4.6)	6 (10.5)	0.17
Flucloxacillin	5 (2.4)	0	0.55
Antidiabetics (Metformin)	3 (1.4)	2 (3.5)	0.61
Co-Illness, n (%)			
Obesity (BMI ≥ 30)	58 (26.9)	20 (35.1)	0.29
Diabetes mellitus II	5 (2.3)	4 (7.0)	0.18
MDRD eGFR ≥ 90 mL/min/1.73 m²	68 (31.2)	24 (42.1)	0.18
MDRD eGFR 60–89 mL/min/1.73 m²	63 (29.2)	15 (26.3)	0.80
MDRD eGFR 30–59 mL/min/1.73 m² (CKD Stage 3)	58 (26.9)	16 (28.1)	0.99
MDRD eGFR 15–29 mL/min/1.73 m² (CKD Stage 4)	25 (11.6)	1 (1.8)	0.02
MDRD eGFR ≤ 15 mL/min/1.73 m² (CKD Stage 5)	2 (0.9)	1 (1.8)	0.50
MRSA infections treated	4 (1.9)	1 (1.8)	1

Continuous variables are presented as mean and median (interquartile range [IQR]). Categorical variables are presented as n (%)

**Table 2:** Target attainment based on vancomycin concentration or AUC_24_ value closest to 120 h after therapy initiation

Target, no (%)	Trough (*n* = 216)	AUC_24_/MIC (*n* = 57)	*p*-value (fisher)	Unadjusted OR [95% CI]
Subtherapeutic^A^	44 (20.4)	12 (21.1)	0.73	1.16 [0.59–2.46]
Therapeutic^B^	119 (55.1)	40 (70.2)	0.049	0.52 [0.27–0.97]
Supratherapeutic^C^	53 (24.5)	5 (8.8)	0.01	3.23 [1.35–9.98]

^A^: Subtherapeutic for Trough is < 10 mg/L and subtherapeutic for AUC_24_ is < 400 mg⋅h/L

^B^: Therapeutic for trough is 10–20 mg/L and therapeutic for AUC_24_ is 400–600 mg⋅h/L

^C^: Supratherapeutic for trough is > 20 mg/L and supratherapeutic for AUC_24_ is > 600 mg⋅h/L

**Fig. 1:**
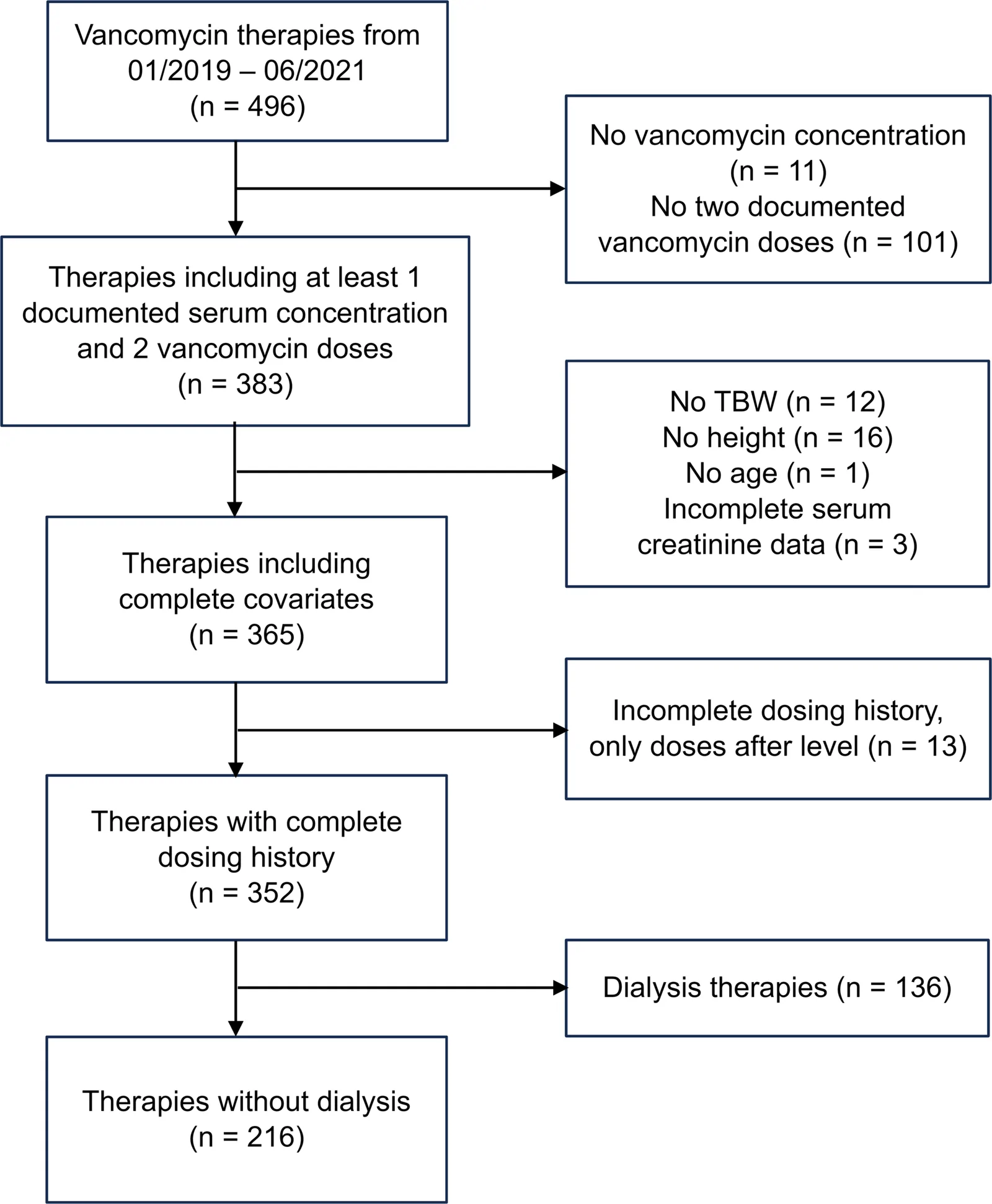
CONSENT Inclusion flowchart for the trough-based-dosing group

**Fig. 2:**
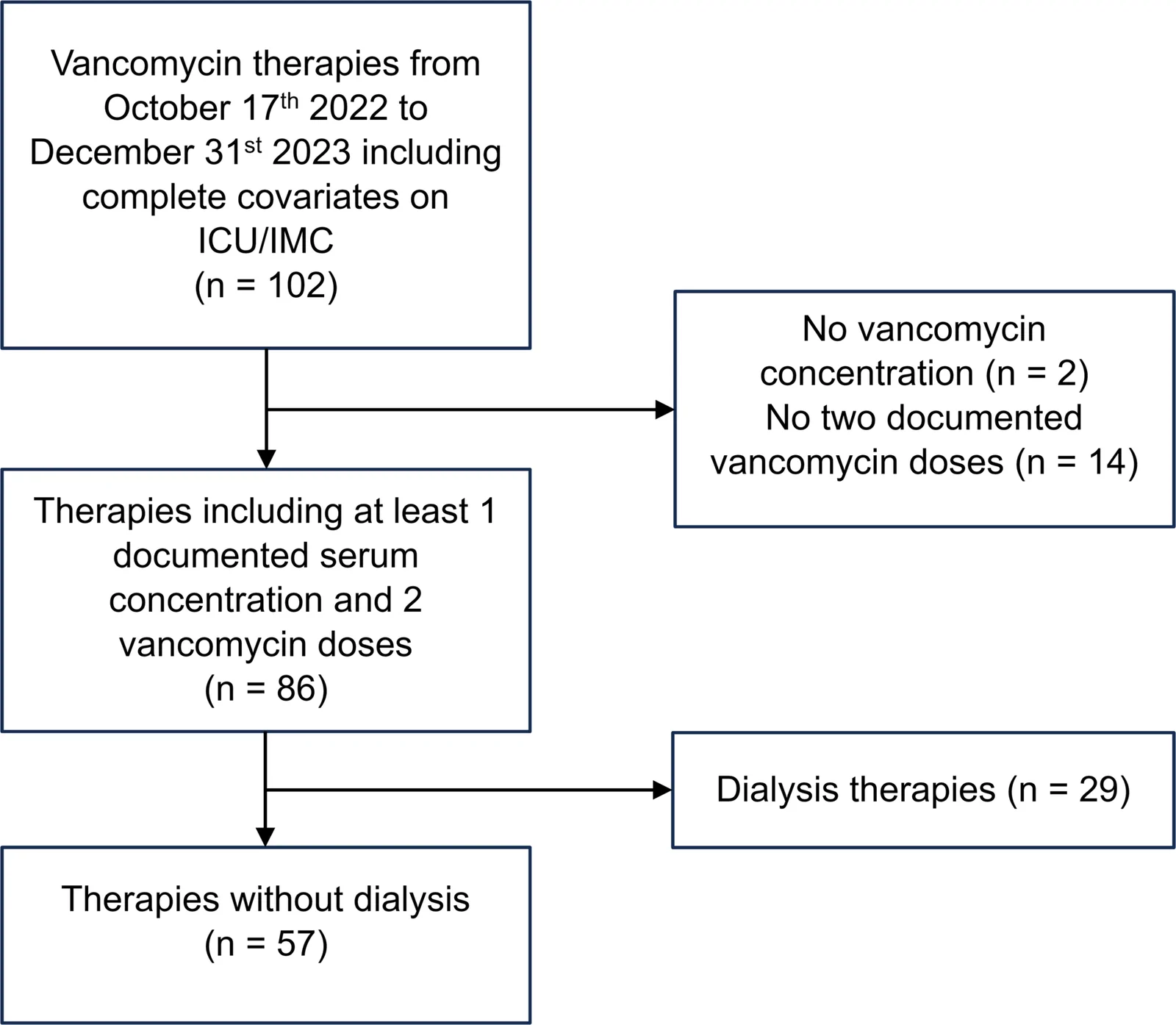
CONSENT Inclusion flowchart for the AUC_24_-based-dosing group

**Fig. 3:**
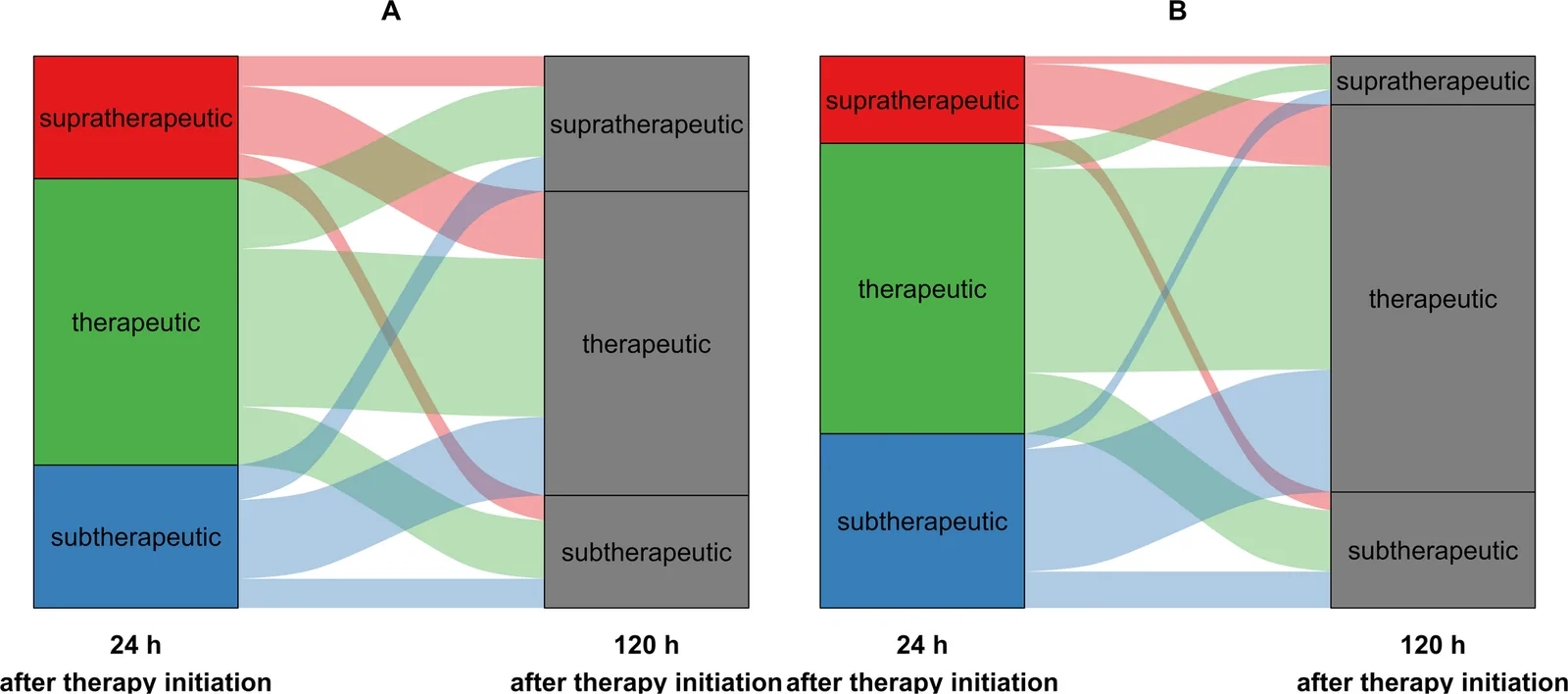
Distribution of vancomycin dosing in subtherapeutic, therapeutic and supratherapeutic ranges in IMC/ICU patients: comparative Sankey diagrams for trough based (**A**) and AUC_24_ based (**B**) dosing strategies, illustrating the flow between ranges from 24 to 120 h after therapy initiation

**Table 3:** Clinical pharmacist recommendations during AUC-guided MIPD group

Recommendations	*n*	%
Total number of recommendations	145	100.0
Dose adjustment recommended	84	57.9
Current dosing recommended	61	42.1
Implementation of recommendations		
Implemented	139	95.9
Not implemented	6	4.1

## Discussion

A pharmacometric AUC_24_-based dosing strategy for vancomycin was implemented at UKW to explore its potential for optimizing therapy not only in MRSA infections but also in a broader range of clinical indications. The analysis focused on renal safety, target attainment rates, and the variability of therapeutic concentrations while considering the role of factors such as concomitant diseases and nephrotoxic medications. The primary outcome, the incidence of AKI, was lower in the prospective AUC_24_-based dosing group than with traditional trough-based dosing of vancomycin (14.0% vs. 27.8%, *p* = 0.04). This difference corresponded to an odds ratio of 2.32. These findings align with previous studies suggesting that supratherapeutic vancomycin concentrations, often observed in trough-based regimens, contribute to nephrotoxicity [19–23]. These findings are also consistent with previous pre-post implementation studies evaluating the transition from trough-guided to AUC-guided vancomycin dosing. Finch et al. reported reduced nephrotoxicity following implementation of Bayesian AUC-guided dosing, whereas Meng et al. primarily demonstrated improved therapeutic target attainment after implementation of a two-point AUC-guided dosing strategy [24, 25]. Similarly, Stocker et al. reported improved target attainment following implementation of a Bayesian therapeutic drug monitoring service [26]. The present study extends these observations by evaluating Bayesian model-informed precision dosing using InsightRX in a real-world cohort predominantly comprising patients with non-MRSA infections. While baseline characteristics between the groups were largely comparable, differences were noted in age and baseline serum creatinine levels. The mean age of the retrospective group was 65.1 years, compared with 57.3 years in the AUC_24_/MIC group, representing a medium effect size according to Cohen’s criteria. Older age is typically associated with a decline in glomerular filtration rate and elevated serum creatinine levels, which likely explains the higher mean baseline serum creatinine observed in the retrospective group [27, 28]. The trough group showed a higher average baseline serum creatinine of 1.24 mg/dl compared with 1.0 mg/dL in the AUC_24_-based dosing group. Additionally, a higher proportion of patients in the trough level-based group had pre-existing impaired kidney function, defined as an MDRD formula eGFR < 60 ml/min/1.73 m² at the start of therapy. Thus, the retrospective group was not only older but was also more likely to have impaired baseline renal function, which is associated with an increased risk of developing AKI [29]. A meta-analysis by Kim et al. found that, while advanced age is not specifically identified as a risk factor for vancomycin-induced renal failure, concomitant kidney disease significantly increases the risk [30]. The elevated serum creatinine levels observed in the retrospective group may indicate an association with an increased risk of AKI. The retrospective group was more frequently exposed to piperacillin/tazobactam (*p* = 0.01), whereas the AUC_24_-based dosing group more frequently received concomitant immunosuppressive therapy (ciclosporin, tacrolimus, and everolimus). This increased risk, together with trough-based dosing, may have contributed to the higher incidence of AKI in the retrospective cohort. A recent study by Venugopalan et al. found no significant relationship between piperacillin/tazobactam exposure and AKI in patients receiving vancomycin while highlighting that mild cases of AKI (KDIGO stages 1 and 2) may not directly related to antibiotic exposure but instead to other mechanisms, such as tubular transporter inhibition [31]. Research investigating this combination in critically ill patients in IMC/ICU settings also indicates an increased risk of AKI, which has been further supported by the findings of the present study [32, 33]. Sex as a potential risk factor is also discussed in the literature. While Filippone et al., and Kan et al. identified female sex as a possible risk factor, a review by Elyasi et al. described male sex as a less commonly reported risk factor [3, 19, 34]. Female sex was associated with a lower risk of AKI in the conducted Cox regression analysis (refer Supplementary table S7). These findings should be interpreted with caution, as substantially fewer women than men were included in both cohorts and female patients generally had better baseline renal function, which may have contributed to the observed association. Obesity is a well-established risk factor that affects drug pharmacokinetics by influencing the volume of distribution owing to altered tissue distribution, clearance of vancomycin, and the dose requirement, as dosing is based on actual body weight [35]. Several reviews indicate that, for these reasons, higher body weight and obesity, which are often associated with an elevated BMI, are risk factors for AKI [3, 19, 30, 36]. This finding is further supported by the Cox proportional hazards regression analysis conducted in the present study. The concomitant administration of nephrotoxic agents, such as loop diuretics and aminoglycosides, has been widely linked to an increased likelihood of AKI in the literature [1, 3, 6, 19, 22]. Despite the lack of statistical significance, the HR of 0.73 (95% CI 0.32–1.62) for AKI in the implemented AUC_24_-guided-dosing suggests a trend towards a reduced risk of AKI compared with a trough-guided dosing strategy. This non-significant result may be attributed to limited sample size and the small number of observed events, underscoring the need for larger studies. To address potential baseline imbalances and reduce confounding, propensity score matching was performed based on all available covariates (sex, total body weight, age, loading doses, serum creatinine, and all recorded concomitant medication), resulting in well-balanced comparison groups. In the matched dataset, the association between dosing strategy and the risk of AKI was reassessed using logistic regression. The OR of 0.74 (95% CI: 0.26–1.9, *p* = 0.54) suggested a potential reduction in the risk of AKI with AUC_24_-based dosing, although the association did not reach statistical significance. These findings indicate that the lower incidence of AKI observed in the primary analysis was not confirmed after adjustment for baseline differences. The lack of statistical significance reflected the substantial differences between the two groups, as well as unequal group sizes and the relatively small size of the prospective cohort. To further evaluate the observed findings, an ITSA was conducted. The ITSA demonstrated immediate and sustained changes following the implementation of AUC-based MIPD, supporting the observed temporal association between the intervention and AKI occurrence. When comparing the present results with previous studies, it should also be considered that differences in AKI definitions may influence the reported incidence of nephrotoxicity. As AKI was assessed according to the KDIGO criteria in the present study, comparisons with studies using alternative definitions should be interpreted with caution. This is consistent with the systematic review and meta-analysis by Abdelmessih et al., which identified differences in AKI definitions as an important source of heterogeneity among studies comparing AUC-guided and trough-guided vancomycin dosing [37]. Exceeding the therapeutic range is associated with an increased risk of AKI [3, 19, 22, 38]. Target attainment was evaluated using the therapeutic target applicable to the retrospective dosing strategy. Accordingly, the retrospective cohort was assessed using measured vancomycin trough concentrations, whereas the prospective cohort was assessed using Bayesian-estimated AUC_24_-values following implementation of AUC-guided therapeutic drug monitoring. This reflects the transition from trough-guided to AUC-guided therapeutic drug monitoring at our institution. Although AUC_24_-values were retrospectively estimated for the pre-intervention cohort, these estimates were not available during routine care and therefore did not represent the basis for clinical dosing decisions. In this study, the therapeutic range was achieved more consistently in the AUC_24_/MIC group, with fewer supratherapeutic concentrations, resulting in a lower incidence of AKI. The proportion of therapies classified as subtherapeutic after 120 h of therapy was comparable between the two groups. In the prospective group, several therapies were either switched to an alternative antibiotic or discontinued, rendering the measured AUC_24_-values no longer relevant to the treatment outcome. The predefined comparison of target attainment was performed 120 h after initiation of vancomycin therapy in order to capture the effect of therapeutic drug monitoring and subsequent individualized dose adjustments during ongoing therapy rather than the initial dosing regimen. Because the study population comprised critically ill patients with heterogenous renal function, the time required to reach steady state differed considerably between patients, which further supports the use of a predefined time point over the assumption of a uniform steady state. Target attainment was nevertheless evaluated at several time points from 24 h onwards, so that its early course, including 48 and 72 h, can be assessed from supplementary figure S3. This is clinically relevant because culture results and antimicrobial stewardship decisions are typically available within the first 72 h of therapy. Differences were observed between the measured vancomycin trough concentrations and the retrospectively calculated AUC values (see Supplementary table S5 and S6). These may be explained by the fact that AUC estimation accounts for both dosing intervals and administered doses. Additionally, the use of a single global model fit based on the complete data set at a single time point may have further influenced the observed values. It is noteworthy that the first vancomycin concentration determination occurred, on average, approximately 27 h after therapy initiation in both the retrospective group (27.6 h) and the prospective group (27.1 h). This suggests that, in the retrospective group, serum concentration measurements were often taken before steady state was reached. Adjusting doses based on serum concentrations obtained before steady state may lead to suboptimal vancomycin dosing, thereby complicating the achievement of the therapeutic range. On average, 4.1 and 5.1 vancomycin concentrations were measured per therapy in the AUC-guided cohort, this corresponds to approximately 0.8 concentration measurements per treatment day in both groups, indicating that implementation of AUC-guided MIPD did not increase sampling frequency relative to treatment duration. Of the 145 documented clinical pharmacist recommendations, 139 (95.9%) were implemented, reflecting a high level of acceptance of pharmacist-led dose recommendations in routine intensive care (refer Table 3).

In line with this, analysis of the retrospective therapies using InsightRX showed that in 137 of the 216 evaluated treatments (63.4%), at least one different dosing recommendation would have been generated. Most treated infections were not MRSA-related, with only 4 cases (1.9%) in the retrospective group and 1 case (1.8%) in the prospective group. The introduction of pharmacometric AUC_24_-based vancomycin dosing improved target attainment and reduced the proportion of supratherapeutic vancomycin exposure, regardless of the underlying pathogen. To date, the optimal target concentration for non-MRSA pathogens has not been well studied. Assuming that lower dosages may be sufficient, toxicity may be further reduced by MIPD. This was reflected in improved adherence to therapeutic target ranges and fewer supratherapeutic concentrations. Although a lower incidence of AKI was observed, this association was not confirmed after adjustments for baseline differences. Given that MRSA infections comprised less than 2% of cases in both groups, these findings support further evaluation of AUC_24_-based dosing in patients with non-MRSA infections. Furthermore, the flexibility of drug level monitoring in routine clinical practice, including the ability to sample blood before achieving steady state, allows for early individualized dose adjustments, further enhancing patient safety. These improvements align with the primary goal of AUC_24_-based dosing: optimizing the safety of vancomycin therapy.

### Limitations

This study has several limitations. First, it was conducted as a single-center study. When interpreting the results, it must also be considered that the implementation of AUC_24_-based MIPD has led to increased attention being paid to vancomycin dosing and potential adverse reactions associated with vancomycin. The presence of a clinical pharmacist in the prospective group, responsible for optimizing dosing and monitoring vancomycin concentrations in active prescriptions in addition to the existing infectious diseases specialist, could introduce a potential bias in therapy monitoring. The clinical pharmacist’s participation is integral to the pharmacometric approach and therefore cannot be eliminated as a limitation. As this was a pre-post-intervention study, temporal changes in clinical practice may also have influenced the observed outcome. In addition, regression to the mean cannot be excluded, as changes in AKI incidence over time may partly reflect natural variation independent of the intervention. In particular, increasing institutional awareness regarding vancomycin dosing and therapeutic drug monitoring following the implementation of the AUC-guided dosing strategy cannot be excluded. Concomitant aminoglycoside therapy was assessed only as a binary variable, and detailed information on dosing regimen, treatment duration, and therapeutic drug monitoring was not available. Although the retrospective cohort included both pre-pandemic and pandemic periods, while the prospective cohort was enrolled during the later phase of the COVID-19 pandemic, the influence of temporal changes in clinical workflows cannot be completely excluded. Both cohorts covered all seasons, with the retrospective cohort spanning more than two years and the prospective cohort more than one year. Therefore, seasonal effects are less likely to explain the observed findings. Moreover, it is important to acknowledge the small sample size of the prospective comparison group. Only treatments administered in intermediate or intensive care units were included to ensure comparability, given that the retrospective group exclusively comprised patients from intermediate and intensive care units. A larger sample size could increase the power to detect differences in the primary endpoint and thereby increase statistical power. No formal a priori sample size calculation was performed because all patients treated with parenteral vancomycin during the study period were included. Furthermore, therapeutic target attainment was assessed using the pharmacokinetic target applicable to the respective therapeutic drug monitoring strategy in each study period. Consequently, different pharmacokinetic endpoints were compared between the retrospective and prospective cohorts. Although AUC_24_-values were retrospectively estimated for the pre-intervention cohort, these estimates were not available for clinical dose adjustments and therefore were not used to define target attainment in the analysis. A systematic hybrid monitoring strategy combining trough- and AUC-based monitoring was not evaluated in this study. Although the SOP provided for trough sampling outside the working hours of the clinical pharmacist, no conclusions can be drawn as to whether a systematic hybrid approach would further improve the clinical pharmacist workflow or reduce monitoring requirements. Retrospective data were extracted from electronic medical records, which may contain inaccuracies. Although a plausibility and completeness assessment were conducted to avoid such issues, they cannot be completely ruled out.

## Conclusion

This study observed a lower incidence of AKI following the implementation of MIPD-AUC_24_-guided dosing for parenteral vancomycin therapies compared with trough-based dosing. Given the differences in baseline characteristics and group sizes, this result should be interpreted with caution. Additionally, AUC_24_-guided dosing enabled therapeutic target attainment significantly more frequently. Moreover, it significantly reduced the number of instances exceeding the therapeutic target range and thereby the risk of toxic effects. These findings suggest that patients with non-MRSA indications may also benefit from AUC_24_-based MIPD and early dose optimization, which may contribute to improved patient and treatment safety. Furthermore, larger studies are needed to determine additional factors influencing these endpoints and to define AUC_24_-target ranges for non-MRSA indications.

## Supplementary Information

Below is the link to the electronic supplementary material.

Supplementary Material 1

Supplementary Material 2

Supplementary Material 3

## Data Availability

The datasets generated and analyzed during the current study are available within the article and its supplementary information files. Due to patient privacy regulations and institutional data protection policies, individual-level data are not publicly available. Anonymized data may be available from the corresponding author upon reasonable request and with permission of the Ethics Committee of Julius-Maximilians-University Wuerzburg.

## Abbreviations

AKI: Acute kidney injury
AUC: Area under the curve
CI: Confidence interval
MIC: Minimum inhibitory concentration
MIPD: Model informed precision dosing
MRSA: Methicillin resistant Staphylococcus aureus
OR: Odds Ratio
SOP: Standard operating procedure
UKW: University Hospital Wuerzburg
