# Supplementary material for: Model-informed AUC-guided dosing for parenteral vancomycin in critically ill patients: impact on target attainment and renal safety – a pre-post intervention study
Source: BMC Pharmacol Toxicol. 2026 Sep 15;27:128. doi: 10.1186/s40360-026-01224-x (PMC13589384; doi:10.1186/s40360-026-01224-x)
Supplement: Supplementary file 2 [file 40360_2026_1224_MOESM2_ESM.docx]

**Electronical Supplement to Model informed AUC based dosing for parenteral Vancomycin reveals beneficial outcomes equally for MRSA and Non-MRSA infections in critically ill patients**

**Supplement Table S1: MRSA Prevalence at University Hospital Würzburg over time**

|  | **Interval** | | | | |
| --- | --- | --- | --- | --- | --- |
| **Pathogen** | 2020 | 2021 | 2022 | 2023 | 2024 |
| Staphylococcus aureus | 136 | 149 | 149 | 128 | 114 |
| Among these: MRSA [%] | 6 (4.4) | 4 (2.7) | 5 (3.4) | 6 (4.7) | 3 (2.7) |

**Supplement Table S2: Antimicrobial resistance Staphylococcus aureus (MRSA) in 2022 in EU^1^**

| **Region** | **Resistant isolates, percentages (%)** |
| --- | --- |
| Austria | 3.9 |
| Belgium | 4.2 |
| Bulgaria | 12.0 |
| Cyprus | 50.8 |
| Czechia | 7.5 |
| Germany | 3.9 |
| Denmark | 1.9 |
| Estonia | 2.2 |
| Greece | 39.0 |
| Spain | 25.8 |
| Finland | 2.3 |
| France | 10.4 |
| Croatia | 31.1 |
| Hungary | 20.9 |
| Ireland | 10.4 |
| Iceland | 2.8 |
| Italy | 29.9 |
| Liechtenstein | - |
| Lithuania | 9.4 |
| Luxembourg | 4.7 |
| Latvia | 7.2 |
| Malta | 19.0 |
| Netherlands | 1.9 |
| Norway | 1.1 |
| Poland | 13.3 |
| Portugal | 25.0 |
| Romania | 38.9 |
| Sweden | 1.9 |
| Slovenia | 8.7 |
| Slovakia | 18.0 |

**Supplement Table S3: Endpoint AKI**

| **Endpoint** | **TROUGH**  **(n = 216)** | **AUC/MIC**  **(n = 57)** | **p-value (fisher)** | **Unadjusted OR [95% CI]** |
| --- | --- | --- | --- | --- |
| AKI, no. (%) | 60 (27.8) | 8 (14.0) | 0.04 | 2.32 [1.08 – 5.59] |

**Supplement Table S4: Achievement of therapeutic target and sub- and supratherapeutic ranges for AUC-based dosing group**

| **AUC range** | **Time [hours]** | | | | | | | | |
| --- | --- | --- | --- | --- | --- | --- | --- | --- | --- |
|  | 24 h | 36 h | 48 h | 60 h | 72 h | 84 h | 96 h | 108 h | 120 h |
| Subtherapeutic  (< 400 mg*h/L) [%] | 31.6 | 28.1 | 31.6 | 31.6 | 26.3 | 21.1 | 22.8 | 21.1 | 21.1 |
| Therapeutic  (400 – 600 mg*h/L)  [%] | 52.6 | 56.2 | 49.1 | 49.2 | 63.2 | 70.2 | 68.4 | 68.4 | 70.2 |
| Supratherapeutic  (> 600 mg*h/L)  [%] | 15.8 | 15.8 | 19.3 | 19.3 | 10.5 | 8.8 | 8.8 | 10.5 | 8.8 |

**Supplement Table S5: Achievement of therapeutic target and sub- and supratherapeutic ranges for trough-based dosing group – trough levels**

| **Level range** | **Time [hours]** | | | | | | | | |
| --- | --- | --- | --- | --- | --- | --- | --- | --- | --- |
|  | 24 h | 36 h | 48 h | 60 h | 72 h | 84 h | 96 h | 108 h | 120 h |
| Subtherapeutic  (< 10 mg/mL)  [%] | 25.9 | 24.5 | 23.6 | 24.1 | 22.7 | 21.3 | 20.4 | 20.4 | 20.4 |
| Therapeutic  (10 – 20 mg/L)  [%] | 51.9 | 51.9 | 51.4 | 50.5 | 52.3 | 54.2 | 55.1 | 52.3 | 55.1 |
| Supratherapeutic  (> 20 mg/L)  [%] | 22.2 | 23.6 | 25.0 | 25.5 | 25.0 | 24.5 | 24.5 | 27.3 | 24.5 |

**Supplement Table S6: Achievement of therapeutic target and sub- and supratherapeutic ranges for trough-based dosing group – AUC-ranges**

| **AUC range** | **Time [hours]** | | | | | | | | |
| --- | --- | --- | --- | --- | --- | --- | --- | --- | --- |
|  | 24 h | 36 h | 48 h | 60 h | 72 h | 84 h | 96 h | 108 h | 120 h |
| Subtherapeutic  (< 400 mg*h/L) [%] | 79.6 | 69.0 | 58.3 | 50.5 | 48.6 | 45.4 | 41.7 | 36.3 | 36.3 |
| Therapeutic  (400 – 600 mg*h/L)  [%] | 18.1 | 25.0 | 34.3 | 38.9 | 39.8 | 41.2 | 42.1 | 44.4 | 41.7 |
| Supratherapeutic  (> 600 mg*h/L)  [%] | 2.3 | 6.0 | 7.4 | 10.3 | 11.6 | 13.4 | 16.2 | 19.0 | 21.8 |

**Supplement Table S7: Cox hazard regression**

| **Variable** | **Hazard ratio (HR)** | **Beta Coefficient** | **Standard Error (SE)** | **95 % Confidence Interval (CI)** | **p-value** |
| --- | --- | --- | --- | --- | --- |
| Dosing method (0 = trough, 1 = AUC) | 0.73 | -0.32 | 0.41 | 0.32 – 1.62 | 0.44 |
| Sex | 0.36 | -1.03 | 0.33 | 0.19 – 0.69 | < 0.01 |
| BMI | 1.06 | 0.06 | 0.02 | 1.02 – 1.10 | < 0.01 |
| Age | 1.02 | 0.02 | 0.01 | 1.00 – 1.04 | 0.12 |
| Vasopressors | 1.76 | 0.57 | 0.39 | 0.81 – 3.82 | 0.15 |
| Loop Diuretics | 1.88 | 0.63 | 0.28 | 1.08 – 3.27 | 0.03 |
| Piperacillin/Tazobactam | 3.24 | 1.18 | 0.33 | 1.71 – 6.15 | < 0.01 |
| Aminoglycoside | 3.27 | 1.19 | 0.49 | 1.26 – 8.50 | 0.01 |
| Immunosuppressants | 2.47 | 0.91 | 0.43 | 1.07 – 5.70 | 0.03 |
| Flucloxacillin | 2.06 | 0.72 | 0.46 | 0.84 – 5.03 | 0.11 |

**Supplement Figure S1: Kaplan-Meier-Estimation of the probability of AKI occurrence in vancomycin therapy: comparison of trough-based and AUC-guided dosing in IMC/ICU-patients at UKW**

**Supplement Figure S2: Target attainment for trough and AUC-based dosing groups after vancomycin therapy initiation**

target attainment trough-based dosing vs. AUC24/MIC

proportion [%]

| time after therapy initiation [hours] | trough subtherapeutic | trough target | trough supratherapeutic | AUC subtherapeutic | AUC target | AUC supratherapeutic |
| --- | --- | --- | --- | --- | --- | --- |
| 24 | 25.9 | 51.9 | 22.2 | 31.6 | 52.6 | 15.8 |
| 36 | 24.5 | 51.9 | 23.6 | 28.1 | 56.2 | 15.8 |
| 48 | 23.6 | 51.4 | 25 | 31.6 | 49.1 | 19.3 |
| 60 | 24.1 | 50.5 | 25.5 | 31.6 | 49.2 | 19.3 |
| 72 | 22.7 | 52.3 | 25 | 26.3 | 63.2 | 10.5 |
| 84 | 21.3 | 54.2 | 24.5 | 21.1 | 70.2 | 8.8 |
| 96 | 20.4 | 55.1 | 24.5 | 22.8 | 68.4 | 8.8 |
| 108 | 20.4 | 52.3 | 27.3 | 21.1 | 68.4 | 10.5 |
| 120 | 20.4 | 55.1 | 24.5 | 21.1 | 70.2 | 8.8 |

**Supplement Figure S3: Interrupted time series analysis of the proportion of therapies with AKI occurende before and after the implementation of AUC-based MIPD for vancomycin**

References

1.	(ECDC) ECfDPaC. Surveillance Atlas of Infectious Diseases - Antimicrobials resistance -Staphylococcus aureus - Meticillin (MRSA) - R - resistant isolates - 2022. Accessed 07.01.2024, 2024.
